# Integrative pan-cancer analysis identifies CCHCR1 as a prognostic biomarker and therapeutic target driving EMT in hepatocellular carcinoma via PI3K/AKT activation

**DOI:** 10.1016/j.gendis.2025.101960

**Published:** 2025-11-28

**Authors:** Shuting Huang, Wanqiu Li, Kaifang Wang, Yu Cai, Lirui Qian, Yiran Liu, Feng Gao, Tong Fang, Kin Yip Tam, Ou Sha

**Affiliations:** a School of Basic Medical Sciences, Shenzhen University Medical School, Shenzhen, Guangdong 518000, China; b Faculty of Health Sciences, University of Macau, Taipa, Macau SAR 999078, China; c School of Dentistry, Shenzhen University Medical School, Shenzhen, Guangdong 518000, China

The development and progression of cancer involve complex interactions among multiple signaling pathways and molecular mechanisms.^1^ The identification and characterization of novel biomarkers with broad clinical utility are essential for elucidating cancer mechanisms. Coiled-coil α-helical rod protein 1 (CCHCR1), identified as a centrosome-associated molecule localized to P-bodies and centrosomes, has been found to be involved in cytoskeletal organization, cell proliferation, and differentiation.^2^^,^^3^ Recent studies suggest its potential oncogenic role in certain cancers; however, its function across a broad range of malignancies and the underlying molecular mechanisms have not been fully characterized. In this study, we conducted an integrated analysis of CCHCR1 in pan-cancers and thoroughly investigated its oncogenic mechanisms both *in vitro* and *in vivo*.

The Cancer Genome Atlas (TCGA) data revealed significant up-regulation of CCHCR1 in 15 types of unpaired and 11 types of paired cancer tissues compared with the respective normal tissues, including liver hepatocellular carcinoma (LIHC) (Fig. 1A and B; Fig. S1A). The Tumor Immune Single-cell Hub (TISCH) database analysis indicated predominant CCHCR1 expression in malignant cells across 16 tumor types, e.g. LIHC cells (Fig. S1B), suggesting a functional role in tumorigenesis. High CCHCR1 expression was associated with poor overall survival (OS) and disease-free survival (DFS) in most cancers, including LIHC, but with a favorable prognosis in rectum adenocarcinoma (READ) (Fig. 1C; Fig. S1C and S1D). These cancer-type-specific associations suggest tissue-dependent functions of CCHCR1. Receiver operating characteristic (ROC) analysis demonstrated high diagnostic accuracy in 5 cancers, with the highest area under the ROC curve (AUC) value of 0.871 in the TCGA-LIHC cohort (Fig. 1D; Fig. S1E). TCGA data showed that CCHCR1 expression was correlated with tumor stage in multiple cancers, including LIHC and other 7 cancers (Fig. 1E). Univariate and multivariate analyses revealed that CCHCR1 could be an independent prognostic biomarker for LIHC patients (Table S1).

**Figure 1:**
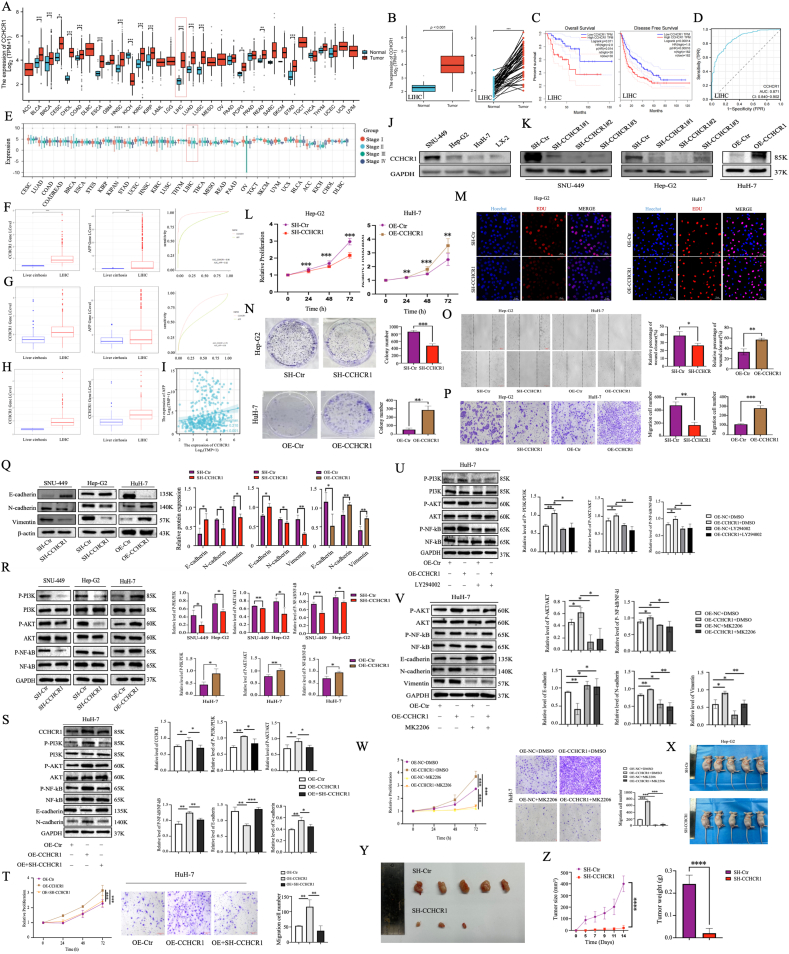
Pan-cancer bioinformatics analysis and experimental validation of CCHCR1 in LIHC. **(A)** Data from the TCGA database demonstrated that CCHCR1 was significantly up-regulated in multiple cancer types, including bladder urothelial carcinoma (BLCA), breast invasive carcinoma (BRCA), cervical squamous cell carcinoma (CESC), cholangiocarcinoma (CHOL), colon adenocarcinoma (COAD), esophageal carcinoma (ESCA), head and neck squamous cell carcinoma (HNSC), kidney renal clear cell carcinoma (KIRC), kidney renal papillary cell carcinoma (KIRP), LIHC, lung adenocarcinoma (LUAD), lung squamous cell carcinoma (LUSC), pheochromocytoma and paraganglioma (PCPG), rectum adenocarcinoma (READ), and stomach adenocarcinoma (STAD). In contrast, CCHCR1 expression in kidney chromophobe (KICH) was significantly higher in normal tissues than in tumor tissues. **(B)** CCHCR1 expression was significantly elevated in tumor samples compared with both unpaired and paired adjacent normal tissues in the TCGA-LIHC cohort. **(C)** High CCHCR1 expression was associated with poor OS and DFS in LIHC. **(D)** ROC curve analysis showed that CCHCR1 had high diagnostic accuracy in LIHC. **(E)** CCHCR1 expression was significantly correlated with the pathological stages of quite a few cancers, including soft tissue sarcoma (STES), the pan-kidney cohort (KICH + KIRC + KIRP, KIPAN), STAD, LIHC, ovarian serous cystadenocarcinoma (OV), testicular germ cell tumors (TGCTs), skin cutaneous melanoma (SKCM), and adrenocortical carcinoma (ACC). **(F, G)** The expression levels of CCHCR1 and AFP were both higher in LIHC than in cirrhosis and the AUC value of CCHCR1 in LIHC was higher than that of AFP in the two datasets. **(H)** In AFP-low expression LIHC tissues, CCHCR1 levels were still significantly higher than those in cirrhosis tissues in both datasets. **(I)** The correlation analysis of AFP and CCHCR1 in LIHC was showed. **(J)** Western blot examined the expression of CCHCR1 among different LIHC and normal LX-2 cells. **(K)** Lentivirus-mediated stable knockdown (KD) and CCHCR1-overexpressing (OE) cell lines were separately generated. **(L)** CCK-8 assay was used to evaluate the effect of CCHCR1 expression on cell proliferation in Hep-G2 and HuH-7 cells. **(M)** CCHCR1-mediated cell proliferation was also assessed using EDU experiments. **(N)** Colony formation assay was further used to validate the impact of CCHCR1 expression on clonogenicity. **(O)** Wound healing assay was performed to assess the effect of CCHCR1 on cell migration. **(P)** Transwell invasion assay was performed to evaluate the invasive ability. **(Q)** The expression of EMT-associated proteins was examined by Western blot. **(R)** The overexpression and knockdown of CCHCR1 respectively increased and decreased the expression levels of P-PI3K, P-AKT, and P–NF-κB in LIHC cells. **(S)** Western blot analysis showing that the re-silencing of CCHCR1 in stable HuH-7 cells overexpressing CCHCR1 (OE + SH-CCHCR1) markedly reduced the phosphorylation levels of PI3K, AKT and NF-κB, and reversed the epithelial–mesenchymal transition (EMT) phenotype, as evidenced by restored E-cadherin expression and decreased N-cadherin levels compared with those in the overexpression group. **(T)** CCK-8 and Transwell assays showing that CCHCR1 re-silencing reversed the proliferation enhancement and abrogated the increased invasion induced by CCHCR1 overexpression. **(U)** Treatment with the PI3K inhibitor LY294002 decreased P-PI3K, P-AKT, and P–NF-κB expression in both control and CCHCR1-overexpressing cells. **(V)** MK2206, a P-AKT inhibitor, reversed the activation of the PI3K/AKT pathway and EMT induced by CCHCR1 overexpression, confirming pathway dependence. **(W)** MK2206 also inhibited the cell proliferation and invasion promoted by the overexpression of CCHCR1. **(X**–**Z)***In vivo* experiments confirmed that the knockdown of CCHCR1 significantly reduced the cancer size and weight in mice.

Alpha-fetoprotein protein (AFP) is the current gold standard for LIHC diagnosis,^4^^,^^5^ but it lacks sensitivity in distinguishing AFP-low LIHC from cirrhosis. In the two GEO datasets (GSE25097 and GSE63898), CCHCR1 showed higher diagnostic accuracy than AFP (Fig. 1F and G). In AFP-low LIHC subgroups, CCHCR1 expression was significantly higher in LIHC than in cirrhosis (Fig. 1H), suggesting that CCHCR1 may serve as a more sensitive diagnostic marker than AFP for distinguishing AFP-low LIHC from cirrhosis. Correlation analysis between CCHCR1 and AFP expression in LIHC showed a weak correlation (*r* = 0.210, Fig. 1I), suggesting that CCHCR1 may serve as an independent prognostic marker and a complementary diagnostic tool to AFP in clinical practice.

To further validate its diagnostic potential, we analyzed datasets including alcoholic and HBV-related hepatitis (GSE83148 and GSE28619). In alcoholic hepatitis, no significant expression differences were observed due to the limited sample size (Fig. S2A and S2B); however, CCHCR1 exhibited higher diagnostic efficacy than AFP (Fig. S2C). In the HBV-related samples, AFP expression showed no significant difference between diseased and normal tissues (Fig. S2D), whereas CCHCR1 was significantly down-regulated and demonstrated superior diagnostic performance (Fig. S2E and S2F). Together, these results suggest that CCHCR1 may have broader and more robust diagnostic potential than AFP across different liver disease contexts.

The cBioPortal analysis revealed CCHCR1 alterations in 2% of cancers, predominantly copy number amplification, with 123 mutation sites (Fig. S3A–S3C). In addition, data from the Gene Set Cancer Analysis (GSCA) database showed an inverse correlation between CCHCR1 methylation and mRNA expression in 31 of 33 cancers, with significant methylation differences in LIHC and 12 other cancers (Fig. S3D and S3E). Furthermore, as shown in Figure S4A, CCHCR1 expression was positively correlated with CD8^+^, CD4^+^ T, and helper T cell infiltrations and negatively correlated with cancer-associated fibroblasts (CAFs) in most cancers, indicating a possible link with adaptive immunity. Conversely, its expression was negatively correlated with neutrophil infiltration in various cancers, suggesting a potential association with reduced innate immune activity that may influence tumor-promoting inflammation and immune evasion. CCHCR1 likely exhibits multifaceted functions across heterogeneous tumor microenvironments. Sangerbox 3.0 analysis showed that CCHCR1 expression was positively correlated with tumor mutational burden (TMB) in 12 and microsatellite instability (MSI) in 13 cancers, both including LIHC (Fig. S4B and S4C). On the other hand, drug sensitivity analysis further identified 28 agents potentially effective in tumors with high CCHCR1 expression (Fig. S4D and S4E).

Based on integrated bioinformatic analysis, CCHCR1 mRNA levels were examined across LIHC cell lines using the HPA database (Fig. S5A). Protein expression analysis showed that CCHCR1 levels were highest in SNU-449, followed by Hep-G2, HuH-7, and normal hepatic LX-2 cells (Fig. 1J). SNU-449 and Hep-G2 were selected for generating stable knockdown cell lines via lentiviral SH-CCHCR1 transduction, while HuH-7 was used to establish a stable overexpression model. Among the three tested shRNAs, SH-CCHCR1#2 demonstrated the highest knockdown efficiency and was selected for subsequent experiments (Fig. 1K).

The CCK-8 assay results showed that CCHCR1 knockdown significantly reduced cell viability in SNU-449 and Hep-G2 cells, whereas overexpression in HuH-7 cells significantly increased viability (Fig. 1L; Fig. S5B). Consistently, EdU and colony formation assays demonstrated that CCHCR1 knockdown suppressed, while overexpression enhanced, LIHC cell proliferation and clonogenic capacity (Fig. 1M and N; Fig. S5C and S5D). Similarly, scratch wound and Transwell assays revealed that CCHCR1 knockdown inhibited, whereas overexpression promoted, LIHC cell migration and invasion (Fig. 1O and P; Fig. S5E and S5F). Western blot analysis demonstrated that CCHCR1 knockdown up-regulated E-cadherin and down-regulated N-cadherin and vimentin, while overexpression produced the opposite effects, indicating its role in promoting epithelial–mesenchymal transition (EMT) (Fig. 1Q).

To elucidate the molecular mechanism of CCHCR1 in LIHC, we investigated several signaling pathways and found that CCHCR1 positively regulates the PI3K/AKT pathway. Western blot analysis showed that CCHCR1 knockdown reduced the phosphorylation of PI3K, AKT, and NF-κB, whereas overexpression increased their phosphorylation (Fig. 1R). To further validate that the oncogenic effects of CCHCR1 are dependent on its expression, a rescue experiment was performed in stable HuH-7 cells overexpressing CCHCR1. Re-silencing CCHCR1 significantly reduced PI3K, AKT, and NF-κB phosphorylation and mitigated the EMT phenotype induced by its overexpression (Fig. 1S). Moreover, CCHCR1 knockdown reversed the enhanced proliferation and invasion observed in CCHCR1-overexpressing HuH-7 cells (Fig. 1T). Treatment with the PI3K inhibitor LY294002 led to a marked decrease in P-PI3K, P-AKT and P–NF-κB levels in both control and CCHCR1-overexpressing cells (Fig. 1U). Similarly, the AKT inhibitor MK2206 suppressed CCHCR1-induced EMT, proliferation, and invasion by reducing P-AKT levels (Fig. 1V and W). These findings demonstrated that CCHCR1 promotes EMT through activation of the PI3K/AKT/NF-κB signaling axis.

For the animal experiments, subcutaneous xenograft models were successfully established using Hep-G2 cells with stable CCHCR1 knockdown. Compared to the control group, CCHCR1 silencing significantly suppressed tumor growth (Fig. 1X–Z), confirming the pro-tumorigenic role of CCHCR1 *in vivo*.

In conclusion, our study demonstrated that CCHCR1 exhibits differential expression across various cancers and is significantly associated with tumor stage and poor prognosis. Importantly, the biological role of CCHCR1 appears to be tumor type-specific, influenced by both the intrinsic molecular background and the tumor microenvironment, and its prognostic significance may vary across cancer types. CCHCR1 shows high diagnostic potential, especially in distinguishing AFP-low LIHC from cirrhosis. Its expression patterns are closely linked to gene amplification and promoter hypomethylation. CCHCR1 may be correlated with the adaptive immune response and influence immunotherapy sensitivity. Functionally, it promotes cancer cell proliferation, migration, invasion, and EMT, particularly in LIHC, through activation of the PI3K/AKT pathway.

## CRediT authorship contribution statement

**Shuting Huang:** Writing – original draft, Validation, Investigation, Formal analysis, Data curation. **Wanqiu Li:** Writing – review & editing, Writing – original draft, Investigation, Formal analysis, Data curation. **Kaifang Wang:** Validation, Supervision. **Yu Cai:** Visualization, Validation. **Lirui Qian:** Visualization, Formal analysis, Data curation. **Yiran Liu:** Validation. **Feng Gao:** Validation, Resources. **Tong Fang:** Validation. **Kin Yip Tam:** Writing – review & editing, Conceptualization. **Ou Sha:** Writing – review & editing, Conceptualization.

## Ethics declaration

This study was approved by the Animal Ethics Committee of Shenzhen University (approval no. IACUC-202500058). All mice used for experiments were provided with a humane living environment.

## Data availability

The data that support the findings of this study are available from the corresponding author upon reasonable request.

## Funding

This study was financially supported by the 10.13039/501100001809National Natural Science Foundation of China (No. 81773939).

## Conflict of interests

The authors have declared that no competing interests exist.
