# Supplementary material for: Integrative pan-cancer analysis identifies CCHCR1 as a prognostic biomarker and therapeutic target driving EMT in hepatocellular carcinoma via PI3K/AKT activation
Source: Genes Dis. 2025 Nov 28;13(5):101960. doi: 10.1016/j.gendis.2025.101960 (PMC13122678; doi:10.1016/j.gendis.2025.101960)
Supplement: Multimedia component 1 [file mmc1.docx]

**Supplementary Materials and methods**

**Materials and Methods**

**mRNA expression analysis of *CCHCR1* in normal human and cancer tissues**

The HPA database was used to assess CCHCR1 mRNA expression in normal human tissues. RNA sequencing datasets for 33 cancer types were obtained from The TCGA database. mRNA expression data for normal tissues were downloaded from the Genotype-Tissue Expression Project (GTEx) database, processed using the STAR alignment pipeline, and reported in transcripts per million (TPM).

**Single-cell expression analysis**

Single-cell mRNA expression of CCHCR1 in various cancer types was analyzed using the TISCH database.

**Survival prognosis analysis in pan-cancers**

Heatmaps and Kaplan–Meier survival curves for OS and DFS were generated using the "Survival Analysis" module of the GEPIA2 database.

**Diagnostic value analysis**

The diagnostic performance of CCHCR1 across various cancers was evaluated by ROC curve analysis using the "pROC" package (version 1.18.0). The area under the ROC curve (AUC) was calculated to determine the diagnostic significance of CCHCR1.

**Clinical stage correlation**

The association between CCHCR1 expression and clinicopathological stage across pan-cancer types in the TCGA cohort was analyzed using the SangerBox database.

**Cox regression analyses**

The univariate and multivariate Cox regression analyses and Kapan-Meier analysis were performed to assess the prognostic value of CCHCR1 in predicting OS in HCC patients using TCGA-LIHC data. The variables with P value <0.05 in univariate Cox regression analysis were selected for further multivariate Cox regression analysis.

**Expression and survival significance of CCHCR1 and AFP in liver diseases**

The mRNA expression and ROC curve analyses of CCHCR1 and AFP in patients with liver cirrhosis and LIHC were conducted using datasets GSE25097 and GSE63898 from the GEO database. The GSE25097 dataset included 40 patients with liver cirrhosis and 268 with LIHC, among whom 134 had low AFP expression. The GSE63898 dataset included 168 patients with liver cirrhosis and 228 with LIHC, including 114 with low AFP expression. Spearman correlation analysis was used to evaluate the relationship between CCHCR1 and AFP expression in LIHC.

To further validate the diagnostic potential of CCHCR1 across different liver disease etiologies, two independent GEO datasets were analyzed, GSE83148, which included 15 alcoholic hepatitis and 7 normal liver samples, and GSE28619, which included 122 HBV-infected and 7 normal liver samples. These datasets were used to compare the expression patterns and diagnostic performance (AUC values) of CCHCR1 and AFP in alcoholic and HBV-related liver diseases.

**Genetic alteration analysis**

Genetic alterations in CCHCR1 were analyzed using the cBioPortal database, which includes data from 10,967 samples across 10,953 patients. The "OncoPrint" module was used to visualize mutation profiles across cancer types. The "Cancer Types Summary" module provided mutation frequencies, while the "Mutations" module identified mutation sites within protein domains.

**Methylation profile of *CCHCR1***

The correlation between CCHCR1 mRNA expression and DNA methylation status in pan-cancers and matched normal tissues was examined using the Gene Set Cancer Analysis (GSCA) database.

**Immune cell infiltration analysis**

The association between CCHCR1 expression and immune cell infiltration in pan-cancers was evaluated using the "Immune" module of the TIMER2.0 database. This analysis incorporated multiple algorithms, including TIMER, EPIC, MCPCOUNTER, CIBERSORT, CIBERSORT-ABS, XCELL, TIDE, and QUANTISEQ.

**Association analysis of *CCHCR1* and TMB/MSI in pan-cancers**

The relationships between CCHCR1 expression and TMB as well as MSI were analyzed separately using the SangerBox database.

**Drug sensitivity analysis**

To evaluate the association between CCHCR1 expression and drug sensitivity, data from the Genomics of Drug Sensitivity in Cancer (GDSC) and the Cancer Therapeutics Response Portal (CTRP) were used to assess drug responses across various cancer types.

**Cell culture**

LIHC cell lines (SNU-449, HuH-7, and Hep-G2) and the normal hepatic cell line LX-2 were obtained from the Shanghai Cell Bank of the Chinese Academy of Sciences. SNU-449 cells were cultured in RPMI-1640 medium (Servicebio, China), while HuH-7, Hep-G2, and LX-2 cells were cultured in high-glucose Dulbecco’s Modified Eagle Medium (DMEM) (Servicebio, China). All media were supplemented with 10% fetal bovine serum (FBS) (Bioagrio, South America) and 1% penicillin-streptomycin (Biosharp, China). Cells were incubated at 37°C in a 5% CO₂ atmosphere. The culture medium was changed every 2–3 days. Cells were passaged at approximately 80% confluence, and those in the logarithmic growth phase were used for subsequent experiments.

**Construction of *CCHCR1* knockdown and overexpressing cell lines**

To establish stable CCHCR1 knockdown cell lines (SNU-449 and Hep-G2), three specific shRNA sequences targeting CCHCR1 were synthesized by GeneCopoeia (Rockville, Maryland, USA). Among these, only one shRNA effectively silenced CCHCR1 expression (5'-GAGCAACTCTCAGACACAGAGAGGA-3') and was used for further experiments. Lentivirus carrying the selected shRNA was generated using a lentiviral expression system (GeneCopoeia). To generate the CCHCR1 overexpressing HuH-7 cell line, a CCHCR1-overexpressing plasmid (pLV3-CMV-CCHCR1-CopGFP-Puro) and the corresponding negative control plasmid (pLV3-CMV-NC-CopGFP-Puro) were obtained from MiaoLingPlasmid, China. Lentiviruses were produced by transfecting these plasmids into 293T cells. A total of 5 × 10⁵ LIHC cells were seeded into six-well plates and infected the following day with the respective lentiviruses expressing CCHCR1 knockdown or overexpression, along with corresponding negative controls. After 24 hours, cells were cultured in puromycin (2 μg/mL; Beyotime, China) for approximately two weeks. A rescue experiment was performed through shRNA-mediated knockdown of CCHCR1 in HuH-7 cells overexpressing CCHCR1. For PI3K/AKT inhibition experiments, 10 µM LY294002/MK2206 (MedChemExpress, US) was added separately to CCHCR1-overexpressing and vector control HuH-7 cells for 24 hours.

**Western blot analysis**

Proteins were extracted using RIPA lysis buffer supplemented with protease inhibitors (MedChemExpress, US). Protein concentrations were determined using a bicinchoninic acid (BCA) assay kit (BOSTER, China). Samples were denatured in SDS-PAGE loading buffer by boiling at 95°C for 3 minutes and cooled on ice for 5 minutes. Proteins were separated on 7.5% or 10% SDS-PAGE gels depending on molecular weight and transferred onto polyvinylidene fluoride (PVDF) membranes using wet transfer at 100V for 60–140 minutes in transfer buffer (25 mM Tris, 192 mM glycine, 20% methanol, pH 8.3). Membranes were blocked with 5% non-fat milk (BD Biosciences, US) at room temperature for 1 hour and incubated overnight at 4°C with primary antibodies. After washing, membranes were incubated with HRP-conjugated anti-rabbit or anti-mouse secondary antibodies (Invitrogen, US). Protein signals were detected using enhanced chemiluminescence (Biosharp, China). The following primary antibodies were used: anti-CCHCR1 (1:500, custom-made), anti-E-cadherin (1:1000, Proteintech, China), anti-N-cadherin (1:1000, Beyotime, China), anti-Vimentin (1:1000, ABmart, China), anti-P-PI3K (1:1000, Cell Signaling, US), anti-PI3K (1:1000, ABmart, China), anti-P-AKT (1:1000, Proteintech, China), anti-AKT (1:1000, Proteintech, China), anti-P-NF-κB (1:1000, Proteintech, China), anti-NF-κB (1:1000, Proteintech, China), anti-GAPDH (1:5000, Proteintech, China), and anti-β-actin (1:5000, Proteintech, China).

**Cell counting kit-8 (CCK8) assay**

Cells were seeded in 96-well plates at a density of 3,000 cells per well in 100 μL of complete medium. Cell viability was assessed at 0, 24, 48, and 72 hours using the CCK-8 assay kit (Servicebio, China). At each time point, 10 μL of CCK-8 reagent was added to each well, including blank controls. Plates were gently shaken and incubated at 37°C in a 5% CO₂ incubator for 1 hour. Absorbance was measured at 450 nm using a microplate reader (EPOCH, BioTek, US).

**EdU assay**

This assay was performed following the manufacturer’s instructions using the 5-Ethynyl-2′-deoxyuridine (EdU) detection kit (Beyotime, China). Cells were seeded into 12-well plates and incubated at 37°C for 24 hours. Subsequently, they were exposed to 10 μM EdU at 37°C for 2 hours, fixed with 4% paraformaldehyde (PFA) (Beyotime, China) at room temperature for 15 minutes, and washed three times with 1 mL of PBS containing 3% BSA, each wash lasting 3–5 minutes. Cells were then permeabilized with 0.3% Triton X-100 and washed three additional times with the same buffer. Afterward, the cells were incubated in the Click Additive solution in the dark for 30 minutes, followed by nuclear staining with Hoechst dye. Microscopic images were acquired to visualize EdU-positive cells. DNA synthesis activity was assessed by calculating the ratio of EdU-positive cells to the total number of cells.

**Colony formation assay**

Cells (1 × 10³) were seeded into six-well plates and cultured under standard conditions (37°C, 5% CO₂). The culture medium was refreshed every three days. After 14 days, the colonies were washed with PBS, fixed with 4% PFA for 15 minutes, and stained with crystal violet (Beyotime, China) for 15 minutes. Colonies, defined as clusters containing ≥50 cells, were quantified using ImageJ software.

**Wound healing assay**

Cells were plated in six-well plates at a density of 8 × 10⁵ cells per well. After 24 hours, a confluent monolayer was formed. A linear scratch was created using a 20 μL pipette tip. Images of the scratch were captured at 0 and 24 hours. Wound width was analyzed using ImageJ software.

**Transwell assay**

Matrigel (Corning, US) was diluted on ice with serum-free medium at a 1:8 ratio. Each Transwell insert (BD Biosciences, US) was coated with 80 μL of diluted Matrigel and incubated at 37°C for 3 hours. Then, 6 × 10^4^ cells suspended in 100 μL of serum-free medium were seeded in the upper chamber. The lower chamber was filled with 500 μL of complete medium to act as a chemoattractant. After 48 hours of incubation at 37°C, the inserts were removed. Non-invading cells on the upper surface of the membrane were removed with a sterile cotton swab. Invading cells on the underside were fixed with 4% PFA for 15 minutes at room temperature and stained with crystal violet (Beyotime, China) for 15 minutes. Stained cells were observed under an optical microscope and quantified using ImageJ software.

**Nude mouse xenograft tumor formation**

Ten male BALB/c nude mice (4–5 weeks old) were obtained from the Guangdong Medical Laboratory Animal Center. After one week of acclimatization, 1 × 10⁶ Hep-G2 cells (stably transfected with SH-Ctr or SH-CCHCR1) were suspended in 100 μL of PBS and subcutaneously injected into the right axilla using a 1 mL syringe (BD Biosciences, US). Injections were performed slowly to ensure even cell distribution. Tumor growth was monitored daily, and mouse health, tumor size, and appearance were recorded. Tumor length (D) and width (d) were measured every two days using calipers, and tumor volume was calculated using the formula: (D × d²)/2. After 14 days, mice were euthanized by CO₂ asphyxiation. Tumors were excised immediately and weighed using an electronic balance. All procedures followed institutional animal ethics guidelines.

**Statistical Analysis**

The expression analysis of CCHCR1 across cancer types was conducted using R software (version 4.2.1). Data visualization was performed using the “ggplot2” package (version 3.3.6). Statistical comparisons were carried out using the “stats” (version 4.2.1) and “car” (version 3.1-0) packages. Biological experiment results are expressed as the mean ± standard error of the mean (SEM). For comparisons between groups, Student’s t-test or one-way analysis of variance (ANOVA) was used. Significance was determined using GraphPad Prism 9 (GraphPad Software) and ImageJ software. Statistical significance was defined as *P < 0.05, **P < 0.01, ***P < 0.001, ****P < 0.0001; “ns” indicated no significance.

**Table 1. The univariate and multivariate COX regression analyses of overall OS in HCC patients from TCGA-LIHC cohort.**

.
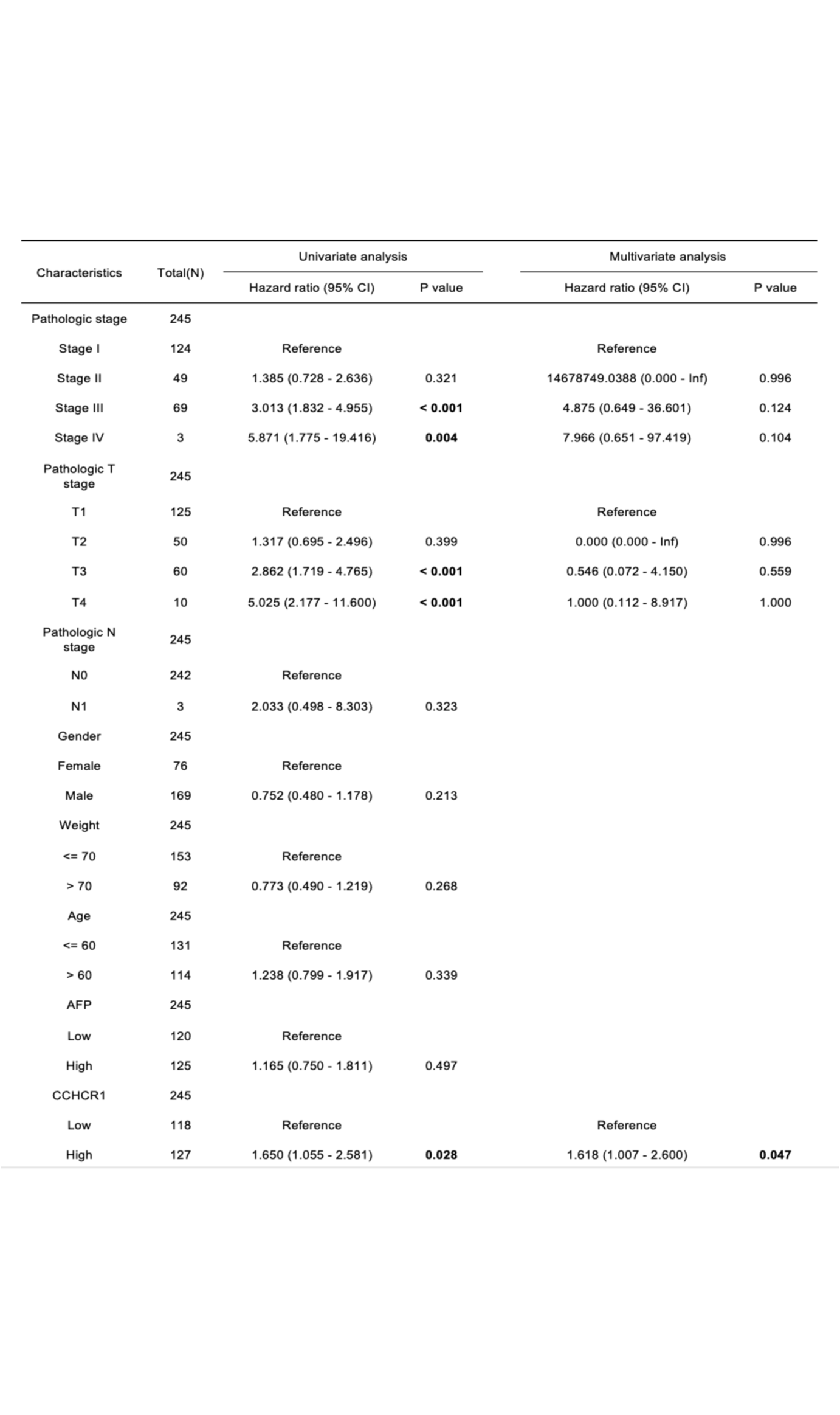


**
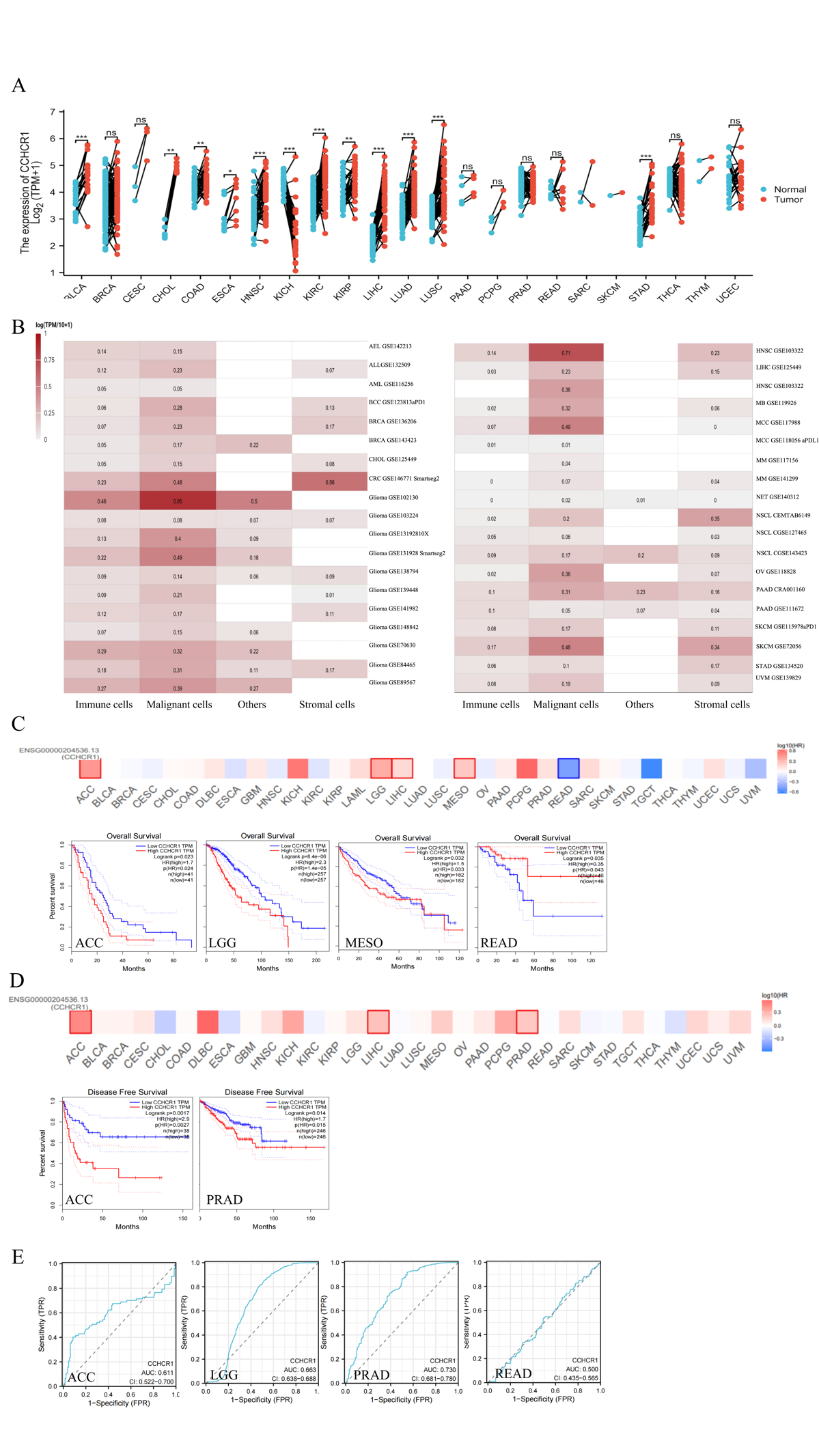
**

**Figure S1** In paired tumor and adjacent normal samples, CCHCR1 was significantly upregulated in BLCA, CHOL, COAD, ESCA, HNSC, KIRC, KIRP, LIHC, LUAD, LUSC, and STAD, but downregulated in KICH (A). Cell-type specific expression patterns of CCHCR1 were analyzed across pan-cancers (B). High CCHCR1 expression was associated with poor OS in ACC, brain lower grade glioma (LGG), LIHC, and mesothelioma (MESO), but with better OS in READ (C). CCHCR1 expression was also significantly associated with DFS, with high levels predicting poor prognosis in ACC, LIHC, and prostate adenocarcinoma (PRAD) (D). ROC curve analysis showed that CCHCR1 had high diagnostic accuracy in ACC, LGG, PRAD, and READ, with area under the AUC values of 0.611, 0.663, 0.730, and 0.500, respectively (E)


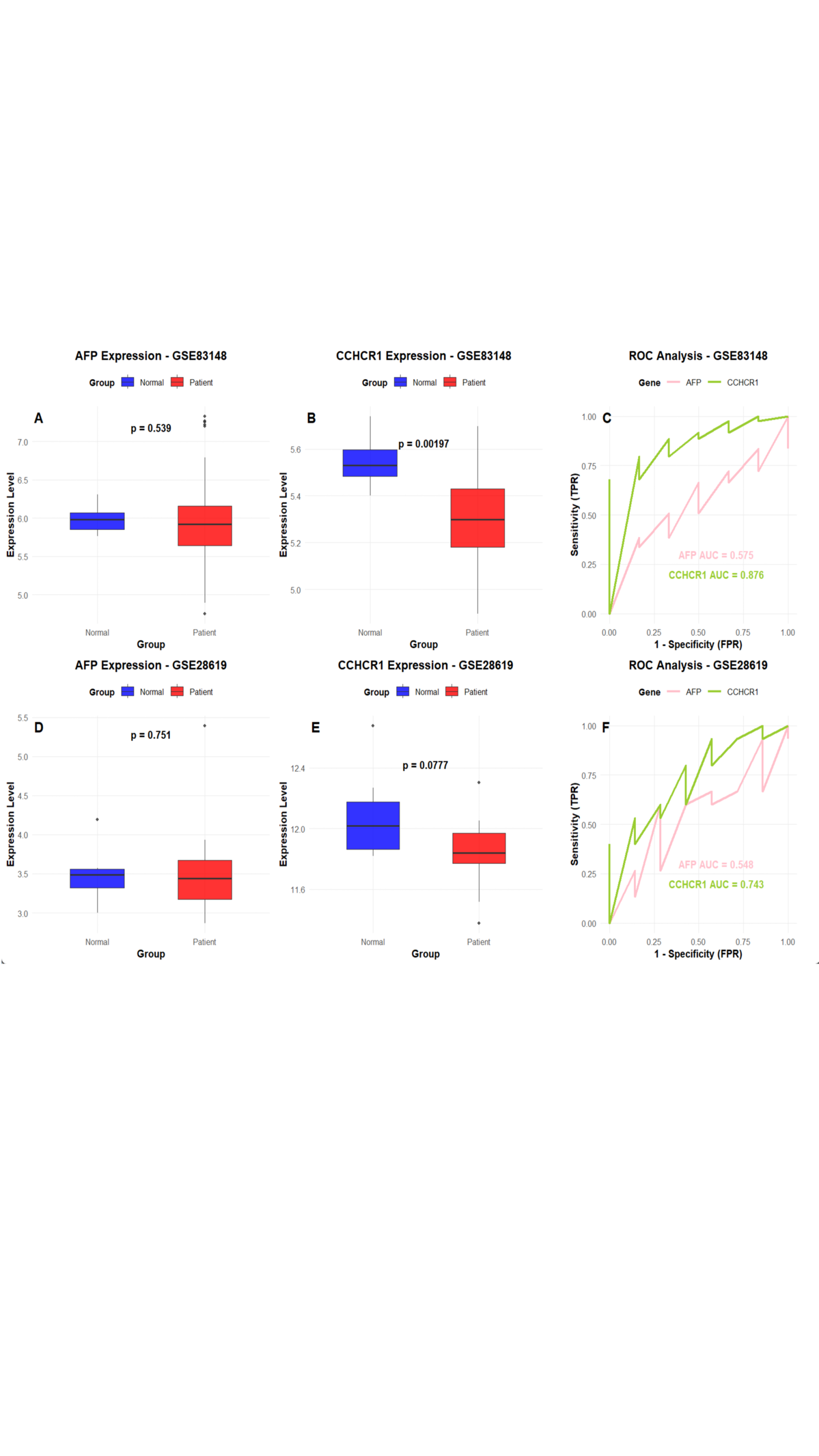


**Figure S2** In alcoholic hepatitis, due to the limited sample size, the expression differences of CCHCR1 and AFP between diseased and normal tissues were not statistically significant; however, CCHCR1 exhibited higher diagnostic efficacy (AUC = 0.743) than AFP (AUC = 0.548) (A-C).

In HBV-related samples, AFP expression showed no significant difference, whereas CCHCR1 was significantly downregulated in HBV-infected tissues and demonstrated superior diagnostic performance (AUC = 0.743 versus 0.548 for AFP) (D-F)


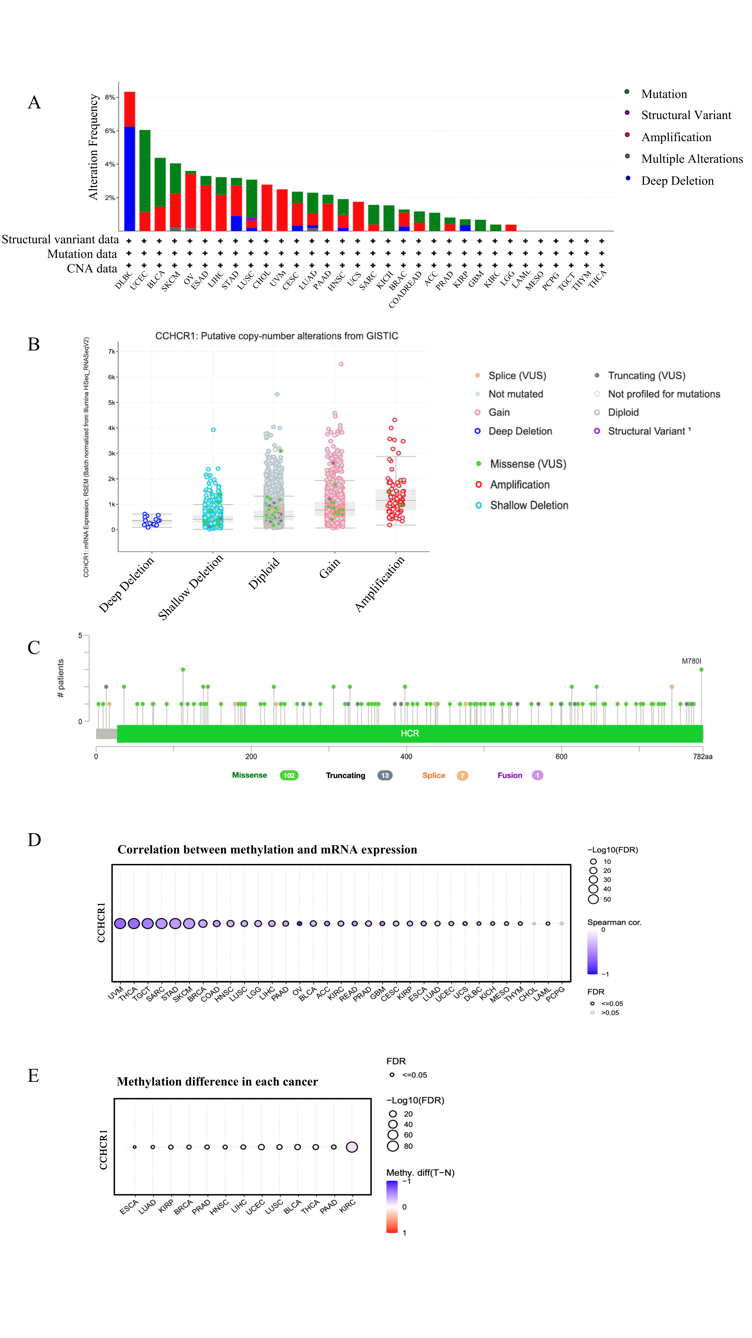


**Figure S3** Comprehensive analysis of genetic alterations and DNA methylation modifications of CCHCR1 across pan-cancer. DLBC exhibited the highest alteration frequency (8.33%), primarily due to deep deletions (A). Copy number amplification was the most common alteration type (B). A total of 123 mutation sites were identified, including 102 missense mutations (82.9%), 13 truncating mutations (10.6%), 7 splice-site mutations (5.7%), and 1 fusion variant (0.8%) (C). The correlation between CCHCR1 mRNA expression and its methylation was significant in 31 out of 33 cancers, with CHOL and PCPG being the only exceptions (D). CCHCR1 methylation difference between cancers and their relevant normal tissues was significant in 13 cancer types, including ESCA, LUAD, KIRP, BRCA, PRAD, HNSC, LIHC, UCEC, LUSC, BLCA, THCA, PAAD and KIRC (E).


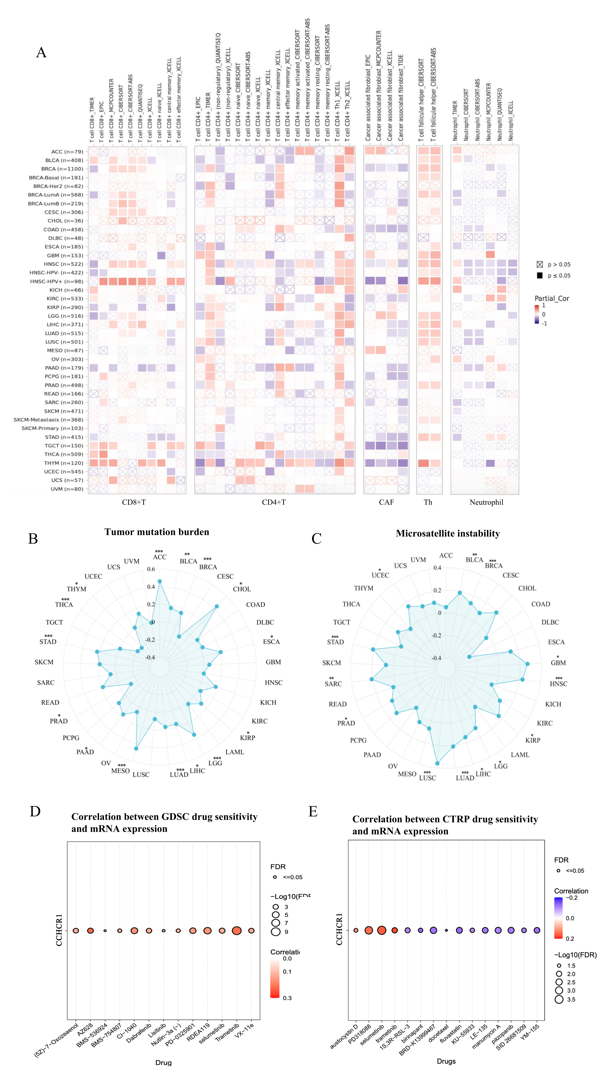


**Figure S4** Analysis of the value of CCHCR1 in immunotherapy and drug sensitivity. CCHCR1 expression was positively correlated with lymphocyte and negatively with CAF and neutriphile infiltration in pan-caners (A). CCHCR1 expression positively correlated with TMB in ACC, BLCA, BRCA, CHOL, ESCA, KIRP, LGG, LIHC, LUAD, MESO, PAAD and STAD, and negatively correlated in PRAD, THCA, and THYM (B). Positive correlations with MSI were observed in BLCA, BRCA, GBM, HNSC, KIRP, LGG, LIHC, LUAD, LUSC, PRAD, SARC, STAD, and UCEC (C). Drug sensitivity analysis based on the GDSC database showed that CCHCR1 expression was significantly associated with increased sensitivity to 13 chemotherapeutic agents, including (5Z)-7-Oxozeaenol, AZ628, BMS-536924, BMS-754807, CI-1040, Dabrafenib, Linsitinib, Nutlin-3a (-), PD-0325901, RDEA119, Selumetinib, Trametinib, and VX-11e (D). Data from the CTRP database revealed significant associations between CCHCR1 expression and responses to 15 drugs, including austocystin D, PD318088, selumetinib, trametinib, 1S,3R-RSL-3, birinapant, BRD-K13999467, docetaxel, fluvastatin, KU-55933, LE-135, manumycin A, pazopanib, SID 26681509, and YM-155 (E).


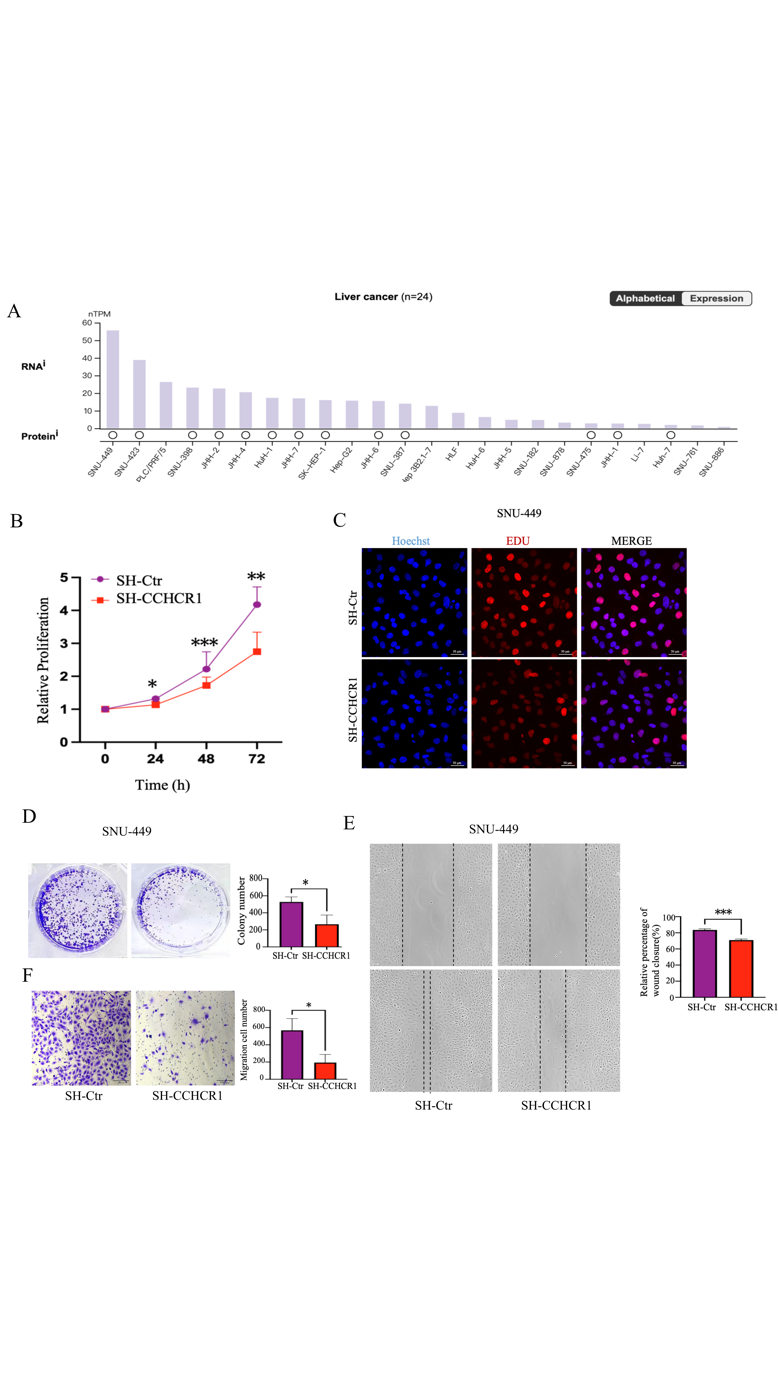


**Figure S5** Functional validation of CCHCR1 in SNU-449 cells. CCHCR1 was analyzed in HPA database and showed different expression levels in various LIHC cell lines (A). CCK-8 assay evaluated the effect of CCHCR1 expression on cell proliferation in SNU-449 cells (B). CCHCR1-mediated cell proliferation was also assessed using EDU experiment (C). Colony formation assay was further used to validate the impact of CCHCR1 expression on clonogenicity (D). Transwell invasion assay was performed to evaluate the invasive ability
